# Non-linear Impact of China's Economic Growth on the Health of Residents—An Empirical Study Based on TVP-FAVAR Model

**DOI:** 10.3389/fpubh.2019.00380

**Published:** 2019-12-20

**Authors:** Fu-Mei He, Tsangyao Chang, Zhen-Jiang Dou, Fangjhy Li, Ke-Chiun Chang

**Affiliations:** ^1^School of Finance, Nanjing University of Finance and Economics, Nanjing, China; ^2^School of Finance, Tongling University, Tongling, China; ^3^Department of Finance, School of Finance, Feng Chia University, Taichung City, Taiwan; ^4^School of Finance, Zhongnan University of Economics and Law, Wuhan, China; ^5^Department of Finance, School of Finance, Hubei University of Economics, Wuhan, China; ^6^School of Economics and Management, Wuhan University, Wuhan, China

**Keywords:** the health of residents, economic growth, consumer spending, high-dimensional macroeconomic data, TVP-FAVAR, D12, I18, O40

## Abstract

This paper uses the 74-dimensional macroeconomic data set from 2005 to 2017 as a sample to construct a TVP-FAVAR model to empirically study the impact of China's economic growth on the health of residents. The study found that China's economic growth has an impact on the health of residents and is transformed into changes in the macroeconomic environment that exhibit non-linear time-varying characteristics. Specifically: (1) During the period of steady economic growth, China's economic growth has caused a significant increase in population mortality rate, infectious disease mortality rate, medical expenses of residents, traffic accident rate, neonatal mortality rate, and tumor mortality rate; (2) During the financial crisis, the positive impact of economic growth on population mortality rate, infectious disease mortality rate, traffic accident rate, and neonatal mortality rate was significantly reduced, while the medical expenses of residents, tumor mortality rate, and cardiovascular morbidity rate and the incidence of mental illness rate has a more obvious inhibitory effect; (3) In the period of sustained economic downturn, the positive impact of economic growth on overall population mortality rate, infectious disease incidence rate, traffic accident rate, and neonatal mortality rate continues to decrease, still negatively affecting the incidence of mental illness rate and cardiovascular morbidity rate. In this paper, we suggested that the Chinese government further promote the transformation of the economic growth model in the new normal economic stage, increase public health fiscal expenditure, and realize an economic development evaluation system that is oriented toward improving the health of residents.

## Introduction

Since the reform and opening-up, China's economic growth has achieved remarkable results and has become the second-largest economy in the world now. As the largest developing country in the world, China's economic type and successful experience are highly concerned by other developing countries. However, while the Chinese economy has achieved remarkable results, the problems of the health of residents are worrying. According to the official research conclusions in 2018, cardiovascular diseases, diabetes, chronic obstructive pulmonary disease, and malignant tumor have become chronic residents of the number one health killer. The fundamental purpose of economic development is to improve the living standards and quality of life of residents. If economic development is at the expense of harming the health of residents, it will form a situation of “putting the cart before the horse.” From the policy level, the Chinese government proposed the “Healthy China 2030” plan in 2016. The ultimate target of this plan is to continuously improve the level of the health of residents, control health hazards and enhance the service capacity of the health industry. Therefore, questions worthy of study are: What is the impact of China's economic growth on the health of residents? What is the transmission mechanism of this effect? In order to answer these questions, first, on the basis of existing research, this paper sorted out the transmission channels of the impact of economic growth on the health of residents, then sets up a high-dimensional time-varying FAVAR econometric model, based on China's data, empirical tests the non-linear impact of China's economic growth on the indicators of the health of residents from a macro perspective, and answers based on research conclusions of the above questions. The structure of this paper is as follows: the first part is the introduction, the second part is the literature review, the third part is the empirical research, and the fourth part is the research conclusions and related policy recommendations.

## Literature Review

### Economic Factors Affecting the Health of Residents Have Become the Focus of Scholars' Research, and Have Formed Abundant Research Results. However, the Question of Whether “Does Richer Mean Healthier?” Remains Controversial

Generally speaking, there are two distinct academic views on the impact of economic growth on the indicators of the health of residents. A literature study concludes that the status of the health of residents and the economy show pro-cyclical characteristics. For example, Brenner (1) took the data of New York State from 1900 to 1967 as a sample. Based on the correlation coefficient analysis method, he found that the economic fluctuation had a negative relationship with the mortality rate of heart disease. That is to say, during the economic recession, the mortality rate of heart disease increased significantly in New York State. Ruhm (2) used data from 1972 to 1991 in the United States as a sample, and empirically studied the relationship between economic conditions and the health of residents based on a fixed-effect model. The results showed that the economic recession was accompanied by a rising unemployment rate, declining income, and deteriorating working environment, which led to increased mental stress, infant mortality rate, and suicide rate. Gerdtham and Ruhm (3) based on data from 23 OECD countries, explored the relationship between macroeconomic development and mortality rate. Their study found that the rising unemployment rate was associated with an increased mortality rate due to cardiovascular diseases, influenza/pneumonia, liver diseases, and traffic accidents. Biggs et al. (4) studied the impact of national economic growth on life expectancy, infant mortality rate and tuberculosis mortality rate in 21 Latin American countries. They also found that public health has pro-cyclical effects, but the intensity of this pro-cyclical effect is disturbed by poverty index and income inequality. Similar studies include Rajan et al. (5).

The conclusion of another literature showed that the health of residents has obvious counter-cyclical effect. For example, Dollar and Kraay (6) found that economic growth tends to lead to income inequality, which in turn has a negative impact on the health of the poor. Ruhm (7) research conclusion showed that the level of physical health and economy has a counter-cyclical relationship. Economic expansion has a higher impact on acute diseases than chronic diseases, while mental health and economy show a pro-cyclical relationship. Noumba (8) estimated the health effect function based on panel data in African countries. The results showed that GNP is an important determinant of the health of residents, but at the same time, he found that richer does not mean healthier. The income gap between the rich and the poor is the main cause of the negative correlation between the two. Ruhm (9), based on the micro-data from 1987 to 2000, found that economic recession will lead to a decrease in smoking and body weight, which will give people more leisure time. The deterioration of economic conditions on lifestyle changes constitutes the counter-cyclical mechanism of the health of residents. Ruhm (10) found that economic expansion significantly increased the incidence of acute myocardial infarction based on data from 20 states in the United States from 1979 to 1998. Hanewald (11) used multiple regression models to reveal the influencing factors of population mortality rate in Germany, and attributed these factors to the macroeconomic, socio-economic and ecological environment, in which economic growth was positively correlated with total mortality rate. In view of the controversy of the research conclusions, some scholars have adjusted the research samples and methods appropriately and reached some compromise conclusions. For example, Reichmuth and Sarferaz (12) used the Bayesian VAR model to validate the relationship between mortality rate, unemployment and economic growth in the United States. Their study found that the youth mortality rate increased with the economic recession, while children and elderly deaths declined with the economic recession. Similar studies included McInerney and Mellor (13).

However, there is a compromise between the business cycle and the health of residents. There is no absolute pro-cyclical or counter-cyclical relationship between the business cycle and the health of residents. In different sample intervals, the correlation between them has time-varying characteristics (14, 15), which implies that with the macroeconomic development. With the change of economic environment, the impact of economic growth on the health of residents may be non-linear. For example, inspired by these predecessors' research conclusions, Lam and Piraerard (16) made a new attempt. First, within the framework of basic analysis of a dynamic general equilibrium model, the non-linear impact of the business cycle on the health of residents was demonstrated, and the conclusions of the theoretical model were simulated. The conclusion showed that in different economic development periods, the impact of economic growth on the health of residents is time-varying. Second, on the basis of theoretical analysis, a time-varying parameter multivariate regression econometric model is set up to empirically test the impact of economic growth on overall population mortality rate, traffic accident rate and cardiovascular morbidity rate in the United States during 1961–2010. The empirical results are in good agreement with the theoretical analysis. That is, under different macroeconomic backgrounds, the United States The correlation between the health of residents and the business cycle is time-varying.

### Scholars Have Also Made Extensive and In-depth Studies on the Transmission Mechanism of the Impact of Economic Development on the Health of Residents

According to different research ideas and purposes, the impact of economic growth on the health of residents can be roughly through the following three transmission channels: First, Ruhm (7, 9) proposed the transmission channel of “time opportunity cost.” The core idea of this theory is that in a good economic development period, giving up work means higher time opportunity cost. Therefore, people will allocate more time to work, less time for fitness, leisure and so on. At the same time, long-term work also means an increase in occupation accidents rate and traffic accident rates. In addition, long-term work can easily increase people's mental stress, which leads to bad habits, such as smoking, alcoholism and so on. Since then, some scholars have carried out a series of empirical studies. Johansson et al. (17) based on Finland's overall data, their study found that during the period of economic expansion, Finland's alcohol consumption increased significantly, accompanied by an increase in alcohol-related mortality rate. Hanewald (11), taking Germany as an example, found that during the period of economic expansion, the consumption of German cigarettes increased significantly, which led to a decline in the level of the health of residents in German. Asfaw et al. (18) based on industry data of U.S. their study found that there is a significant correlation between the incidence of industrial injury rate and the business cycle, in which the mining, construction, and manufacturing industries have obvious pro-cyclical characteristics. De Goeij et al. (19) reviewed the impact of the financial crisis on alcohol consumption systematically. Second, the transmission mechanism of medical security of residents: Brenner (1), Dooley et al. (20), Colman and Dave (21), and other scholars believe that, on the one hand, the economic recession is accompanied by an increase in unemployment rate, which leads to a decrease in disposable income and constraints on medical expenditure. In this case, the health care of residents is not effectively protected. This leads to a decline in overall health. On the other hand, during the economic depression, the government or private sector will reduce the investment in medical facilities, which hinders the development of social medical supply capacity and overall medical level. Third, the transmission mechanism of ecological environment damage: Kahn and Kotchen (22) based on US interstate data found that during the period of rising unemployment rate, people's attention to climate and environment declined, which led to the reduction of environmental protection policies of government departments and the weakening of awareness of ecological environment protection, which had a negative impact on the living environment of residents. Fischer and Heutel (23) introduced pollution variables into the business cycle model and found that pollutant emissions showed pro-cyclical characteristics. Heutel and Ruhm (24), taking the United States as the research object, but the overall mortality rate, three major environmental pollution indicators (carbon monoxide concentration) and economic growth indicators into the framework of the econometric model. Their study found that economic growth led to the increase of environmental pollution indicators, which led to the pro-cyclical characteristics of the overall mortality rate. Chen et al. (25) empirically studied the spatial spillover effects of air pollution in 116 cities of China using the spatial Durbin model. The results showed that sulfur dioxide emissions are positively correlated with lung cancer and respiratory mortality rate.

From the existing research, economic growth is an important factor that causes a change in the level of the health of residents. The time opportunity cost formed by the business cycle, the change of ecological environment brought by economic development and the influence of economic growth on medical care expenditure constitute the main transmission mechanism of economic development on the health of residents. All these provide a certain preliminary basis for the study of this paper. However, there are still some improvements and extensions in the following aspects: First, there are few studies on China to analyze the impact of economic growth on the health of residents. China is currently the second-largest economy in the world. It plays the role of “world factory” in the global economic system and is an important manufacturing power in the world. Therefore, comparing with other countries, taking China as a research sample has important “typical” significance, and has certain reference and enlightenment value for countries with manufacturing industry as the main economic development in the world. Second, as mentioned above, a few recent works of the literature showed that with the change of the macroeconomic environment, the relationship between economic growth and the health of residents has potential non-linear characteristics. At present, China's economy is in a transitional stage. These conclusions are of great enlightenment value to the study of China's economic growth and the health of residents. However, the existing empirical literature mostly adopts fixed coefficient models in the choice of research methods. Although these fixed coefficient models can identify the causal relationship between initial economic growth and the health of residents, they cannot describe the non-linear characteristics of the causal relationship.

Based on the description above, this paper takes China as the research object and sets a TVP-FAVAR econometric model with high-dimensional data to empirically test the non-linear impact of China's economic growth on the health of residents. It is easy to see that this study is a further supplement and extension of the existing research.

## Test Of Non-Liner Relation Between Economic Growth And The Health Of Residents In China

Lam and Piérard (16) introduced the indicators of the health of residents into the endogenous economic growth model and theoretically demonstrated that with the change of macroeconomic environment, the impact of economic growth on the health of residents presents non-linear time-varying characteristics. In contrast, based on the principle of data speaking, this paper constructs a bivariate DCC-GARCH model with China's economic growth and the health of residents, using Jones and Olson (26) research ideas for reference, trying to preliminarily test the possible non-linear relationship between the two.

**Figure 1:**
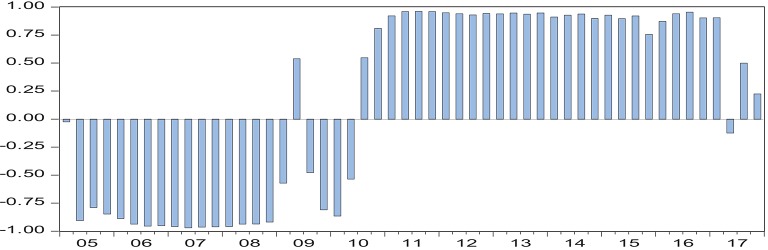
Dynamic correlation coefficient between China's economic growth and the health of residents^1^.

From Figure 1, it can be seen that the correlation coefficient between China's economic growth and the health of residents shows obvious non-linear characteristics in the sample period. As far as the interval difference is concerned, the correlation coefficient between China's economic growth and the health of residents is basically below 0 during the period of 2005 Q1-2010 Q2, which indicates that there is a reverse relationship between China's economic growth and the health of residents during the period of 2010 Q3-2017 Q4. Meanwhile, the correlation coefficient between them is above 0, which indicates that during this period, the relationship between China's economic growth and the health of residents is in the same direction. The empirical results above are in good agreement with reality. During the period of 2005 Q1-2010 Q2, China's economy experienced a period of steady development and the global financial crisis in 2008. According to the time opportunity cost theory (9) mentioned above, under this macroeconomic background, people will choose more time to work, rather than to strengthen health exercise. The outbreak of the financial crisis has a negative impact on economic operation, which is bound to be accompanied by an increase in unemployment. In addition, during this period, the main driving force of China's economy came from the investment of fixed assets, which posed a serious threat to the ecological environment. These factors overlap with each other, leading to a decline in the level of the health of residents. During the period of 2010 Q3-2017 Q4, China's economic growth declined markedly, and the economy entered a recession. In order to cope with the unfavorable situation of economic operation, the Chinese government implemented the supply-side reform, trying to adjust the economy from the supply-side, and the innovative-driven economic growth model achieved initial results, also according to the time opportunity cost. Theoretically, in this macroeconomic context, people will allocate more time to exercise and leisure, and thus improve their overall health. Figure 1 also shows that the correlation coefficients between China's economic growth and indicators of the health of residents are different in different periods within the same time interval. For example, in the first quarter of 2005, 2006, 2008, 2009, and 2010, the correlation coefficients are −0.0245, −0.8865, −0.9577, −0.5718, and −0.8647, i.e., the correlation coefficients are −0.0245, −0.8865, −0.9577, −0.5718, and −0.8647, respectively. The non-linear dynamic correlation between China's economic growth and the health of residents is time-varying. Although the empirical results based on DCC-GARCH model showed that there is a potential time-varying correlation between China's economic growth and the level of the health of residents, the above empirical results cannot describe the dynamic impact and transmission mechanism of China's economic growth on the level of the health of residents. Therefore, this paper will establish a non-linear econometric model for further testing.

## An Empirical Study On Non-Linear Impact Of China's Economic Growth On The Health Of Residents

### Selection of Econometric Model

Since Sims (27) first proposed the vector autoregression (VAR) model, this model has been widely used in the economic field. This is due to the obvious improvement of the model compared with the previous structural equation, without considering the endogenous problem of the model. In addition, the model includes the lag period of variables, which can effectively describe the dynamic impact effect of model variables. However, the early VAR model is facing a series of challenges, such as “information omission,” “liquidity mystery,” and “dimension curse,” which seriously restrict the further use of the model. To solve these problems, some econometric economists have improved the model. Generally speaking, the model is expanding in two directions: one is to expand the information set of model variables, among which Bernanke et al. (28) introduced Stock and Watson (29) dynamic factor model into VAR, and constructed factor-enhanced vector autoregression (FAVAR) model to effectively overcome the shortcomings of traditional small-scale VAR information omission. Another, the traditional fixed coefficient VAR model is extended to a non-linear VAR model, which improves the explanatory power of the VAR model to reality. According to the literature, the non-linear VAR model mainly includes threshold class (STR-VAR), Markov mechanism transfer class (MS-VAR), and time-varying parameter class (TVP-VAR). Compared with the previous two kinds of models, the former two kinds of bases are mainly used. This principle describes the non-linear characteristics mainly through the structural variations of one or several variables in the model, and the time-varying parameter class can effectively capture the non-linear characteristics caused by the transformation of the macroeconomic environment by setting the coefficients as a random walk (30). In recent years, Korobilis (31) has merged two directions of the traditional VAR model and constructed the TVP-FAVAR model, which greatly improves the adaptability of the model to the realistic description.

According to the transmission mechanism mentioned above, the transmission process of China's economic growth on the health of residents involves many variables, such as unemployment rate, medical expenditure, ecological environment, etc. Obviously, the traditional small-scale VAR model cannot meet the needs of this study^2^. In addition, the validation results based on the DCC-GARCH model show that there is a potential time-varying relationship between China's economic policy and the health of residents.

### Econometric Model Setting

According to Korobilis (31), the TVP-FAVAR model is set as follows:

(1) $$\begin{array}{r} X_{t}=\lambda_{t}^{f}F_{t}+v_{t},\;v_{t}:N\left(0,\;V_{t}\right) \end{array}$$

(2) $$\begin{array}{r} \left[\begin{array}{c} F_{t}\\ Y_{t} \end{array}\right]=C_{t}+B_{t,1}\left[\begin{array}{c} F_{t-1}\\ Y_{t-1} \end{array}\right]+L+B_{t,p}\left[\begin{array}{c} F_{t-p}\\ Y_{t-p} \end{array}\right]+\varepsilon_{t},\;\varepsilon_{t}:N\left(0,\;Q_{t}\right) \end{array}$$

*X*_*t*_ is a set of *n* × 1 dimensional macroeconomic information that affects the health of residents of China, including the indicators of the health of residents, of course. The common factor unobservable factor of *l* dimension extracted by *F*_*t*_ from large data set *X*_*t*_ is the common factor load, $\lambda_{t}^{f}$ is the common factor load, *Y*_*t*_ is the index of China's economic growth, *C*_*t*_ is the constant term, *B*_*t,j*_, *j* = 1*L*, is the lag coefficient, *p* is the lag order, ε_*t*_ is the (*n*+1) × 1 error term to indicate the common impact. Subscript *t* denotes that factor load, dynamic lag coefficient and disturbance term are all non-linear time-varying terms. The lag coefficients of the model overlap $\beta_{t}={\left(C_{t},\;vec{\left(B_{t,\;1}\right)}^{{}^{\prime }},\;vec{\left(B_{t,\;2}\right)}^{{}^{\prime }},\;L,\;vec{\left(B_{t,\;j}\right)}^{{}^{\prime }}\right)}^{{}^{\prime }}$ describes the time- varying of lag coefficient and common cause load by first-order random walk.

(3) $$\begin{array}{r} \lambda_{t}=\lambda_{t-1}+v_{t},\;v_{t}:N\left(0,\;W_{t}\right) \end{array}$$

(4) $$\begin{array}{r} \beta_{t}=\beta_{t-1}+\eta_{t},\;\eta_{t}:N\left(0,\;R_{t}\right) \end{array}$$

The impulse response function based on (2) is the non-linear effect of China's economic growth on the health of residents.

From the above model settings, there are six parameters to be estimated, which are β_*t*_, λ_*t*_, *V*_*t*_, *W*_*t*_, *Q*_*t*_, and *R*_*t*_. For ease of expression, let's make θ_*t*_ = (λ_*t*_, β_*t*_) here. Because the stochastic characteristics of β_*t*_ and λ_*t*_ obey linear Gauss, their estimates can be obtained by using the commonly used linear Kalman filter in literature; the exponential weighted moving average method is used to estimate the parameters *V*_*t*_ and *Q*_*t*_; and the forgetting factor (FF) algorithm is used to estimate the parameters *W*_*t*_ and *R*_*t*_ according to Koop and Korobilis (32). The specific steps are as follows:

Step 1: Assign the initial value λ_0_, β_0_, *V*_0_, *Q*_0_ of the parameters to be estimated as follows:

$$\lambda_{0}:N(0,\;\Sigma_{0|0}^{\lambda }),\;\beta_{0}:N\left(0,\;\Sigma_{0|0}^{\beta }\right),\;V_{0}=1\times I_{n},\;Q_{0}=1\times I_{s+1}$$

Step 2: Based on the given initial value and $Z_{t}={\bar{Z}}_{t}$, the predicted state of the parameter λ_0_, β_0_, *V*_0_, *Q*_0_ can be obtained by the filter principle, where ${\bar{Z}}_{t}=\left(Y_{t},\;{\bar{F}}_{t}\right)$, ${\bar{F}}_{t}$ is the common factor extracted from the macroeconomic data set by the principal component analysis method, according to the Kalman filter:

$$\begin{array}{l} \lambda_{t}|\;Data_{1:t-1}:N\left(\lambda_{t|t-1},\;\Sigma_{t|t-1}^{\lambda }\right),\;\\ \;\beta_{t}|Data_{1:t-1}:N\left(\beta_{t|t-1},\;\Sigma_{t|t-1}^{\beta }\right) \end{array}$$

where $\lambda_{t|t-1}=\lambda_{t-1|t-1},\;\Sigma_{t|t-1}^{\lambda }=\Sigma_{t-1|t-1}^{\lambda }+{\hat{W}}_{t},\;\Sigma_{t|t-1}^{\beta }=\Sigma_{t-1|t-1}^{\beta }+{\hat{R}}_{t}$, the error covariance term here is estimated by the forgetting factor algorithm.

${\hat{W}}_{t}=\left(1-k_{3}^{-1}\right)\Sigma_{t-1|t-1}^{\lambda },\;{\hat{R}}_{t}=\left(1-k_{4}^{-1}\right)\Sigma_{t-1|t-1}^{\beta }$, we can see that the parameters *k*_3_ and *k*_4_ control the time-varying forgetting degree of ${\hat{W}}_{t}$ and ${\hat{R}}_{t}$, respectively. Therefore, *k*_3_ and *k*_4_*k*_4_ are called forgetting factors. Lower *k*_3_ or *k*_4_ means that the observation error at *t*−1 time occupies the corrected time error covariance. For example, when *k*_3_ = *k*_4_ = 1 and *W*_*t*_ = *R*_*t*_ = 0, the estimated parameters λ_*t*_ and β_*t*_ are constant.

Step 3: Estimate the parameters *V*_*t*_ and *Q*_*t*_ with EWMA algorithm as follows:

$$\begin{array}{l} {\hat{V}}_{i,t}=k_{1}V_{i,t|t-1}+\left(1-k_{1}\right){\hat{\mu }}_{i,t}{\hat{\mu }}_{i,t}^{\prime }\\ {\hat{Q}}_{i,t}=k_{2}Q_{i,t|t-1}+\left(1-k_{2}\right){\hat{\varepsilon }}_{i,t}{û}_{i,t}^{\prime } \end{array}$$

where ${\hat{\mu }}_{i,t}=x_{i,t}-{\bar{z}}_{t}\lambda_{i,t|t-1},\;i=1,\;L\;n,\;{\bar{\varepsilon }}_{t}={\bar{z}}_{t}-{\bar{z}}_{t-1}\beta_{t|t-1}$, where *k*_1_ and *k*_2_ are decay factors of EWMA, and their properties are similar to *k*_3_ and *k*_4_.

Step 4: Based on the information acquired at *t* time, Kalman filter is used to update the elements in the parameter $\lambda_{t.}\;{\lambda_{t}|Data}_{1:t}:N\left(\lambda_{i,t|t},\;\Sigma_{ii,t|t}^{\lambda }\right),\;i=1,L\;n,$ where $\lambda_{i,t|t}\;=\;\lambda_{i,t|t-1}+\Sigma_{ii,t|t-1}^{\lambda }{\bar{z}}_{t}^{\prime }{\left({\hat{V}}_{ii,t}+{\bar{z}}_{t}\Sigma_{ii,t|t-1}^{\lambda }\bar{z}\prime_{t}\right)}^{-1}\left(x_{t}-{\bar{z}}_{t}\lambda_{t|t-\;1}\right)$

$$\Sigma_{ii,t|t}^{\lambda }=\Sigma_{ii,t|t-1}^{\lambda }-\Sigma_{ii,t|t-1}^{\lambda }{{\bar{z}}^{\prime }}_{\;t}({\hat{V}}_{ii,t}+{\bar{z}}_{t}\Sigma_{ii,t|t-1}^{\lambda }{{\bar{z}}^{\prime }}_{\;t}){\;}^{-1}{\bar{z}}_{t}\Sigma_{ii,t|t-1}^{\lambda }$$

Similarly, update β_*t*_, $\beta_{t}|Data_{1:t-1}:N\left(\beta_{t|t},\;\Sigma_{t|t}^{\beta }\right),\;i=1,\;L\;n$ based on the information acquired at time *t*, where $\beta_{t|t}\;=\;\beta_{t|t-1}+\;\Sigma_{t|t-1}^{\beta }{\bar{z}}_{t-1}^{\prime }{\left({\hat{Q}}_{t}+{\bar{z}}_{t-1}\Sigma_{t|t-1}^{\beta }{\bar{z}}_{t-1}^{\prime }\right)}^{-1}\left({\bar{z}}_{t}-{\bar{z}}_{t-1}{\hat{\beta }}_{t|t-\;1}\right)$

$$\begin{array}{l} \Sigma_{t|t}^{\beta }=\Sigma_{t|t-1}^{\beta }-\Sigma_{t|t-1}^{\beta }\;{\overset{\bar{z}}{}}_{t-1}^{\prime }{\left({\hat{Q}}_{t}+{\bar{z}}_{t-1}\Sigma_{t|t-1}^{\beta }{\bar{z}}_{t-1}^{{}^{\prime }}\right)}^{-1}{\bar{z}}_{t-1}\Sigma_{t|t-1}^{\beta } \end{array}$$

Step 5: Run the recursive algorithm, smoothly estimate λ_*t*_, β_*t*_, *V*_*t*_, and *Q*_*t*_ from *t* = *T* − 1, *L* 2, 1 time, and correct them with the real observation value of *t*+1. For λ_*t*_: λ${}_{i,t}|Data_{1:T}:\;N\left(\lambda_{i,t|T},\;\Sigma_{ii,t|T}^{\beta }\right),\;i=1,\;L\;n$, where

$\lambda_{i,t|T}=\lambda_{i,t|t}+C_{t}^{\lambda }\left(\lambda_{i,t+1|T}-\lambda_{i,t+1|t}\right),\Sigma_{ii,t|T}^{\lambda }=\Sigma_{ii,t|t}^{\lambda }{\;+\;C}_{t}^{\lambda }\left(\Sigma_{ii,t|T}^{\lambda }-\Sigma_{ii,t+1|t}^{\lambda }\right)C_{t}^{\lambda }$, where

$C_{t}^{\lambda }=\Sigma_{ii,t|t}^{\lambda }{\left(\Sigma_{ii,t+1|t}^{\lambda }\right)}^{-1}$, for β_*t*_, $\beta_{t}|Data_{1:T}:\;N\left(\beta_{t|T},\;\Sigma_{t|T}^{\beta }\right),\;i=1,\;L\;n$. Where

$$\begin{array}{l} \beta_{t|T}=\beta_{t|t}+C_{t}^{\beta }\left(\beta_{t+1|T}-\beta_{t+1|t}\right),\Sigma_{t|T}^{\beta }=\Sigma_{t|t}^{\beta }\;\\ \;+\;{\;C}_{t}^{\beta }\left(\Sigma_{t+1|T}^{\beta }-\Sigma_{t+\;1|t}^{\beta }\right)C_{t}^{\beta },\\ \beta_{t|T}=\beta_{t|t}+C_{t}^{\beta }\left(\beta_{t+1|T}-\beta_{t+1|t}\right),\Sigma_{t|T}^{\beta }=\Sigma_{t|t}^{\beta }\\ \;+\;C_{t}^{\beta }\left(\Sigma_{t+1|T}^{\beta }-\Sigma_{t+1|t}^{\beta }\right)C_{t}^{\beta },\\ \beta_{t|T}=\beta_{t|t}+C_{t}^{\beta }\left(\beta_{t+1|T}-\beta_{t+1|t}\right),\Sigma_{t|T}^{\beta }=\Sigma_{t|t}^{\beta }\\ \;+\;C_{t}^{\beta }\left(\Sigma_{t+1|T}^{\beta }-\Sigma_{t+1|t}^{\beta }\right)C_{t}^{\beta },C_{t}^{\beta }=\Sigma_{t|t}^{\beta }{\left(\Sigma_{t+1|t}^{\lambda }\right)}^{-1},\\ \text{for }V_{t}\text{ and}\\ Q_{t},V_{t|t-1}^{-1}=k_{1}V_{t|t}^{-1}+\left(1-k_{1}\right)V_{t+1|t+1}^{-1},Q_{t|t-1}^{-1}=k_{2}Q_{t|t}^{-1}\\ \;+\;\left(1-k_{2}\right)Q_{t+1|t+1}^{-\;1}. \end{array}$$

After the five steps mentioned above, the parameters of the model are estimated.

### Variable Description and Processing

The key variables in the model include economic growth, the health of residents and macroeconomic data sets. As far as economic growth is concerned, we adopt the common practice in the literature and take GDP as the proxy index of economic growth over the same period of time. Referring to Ruhm (7, 9), the indicators of the health of residents in this paper mainly include: population mortality rate, infectious disease incidence rate, medical expenses of residents, neonatal mortality rate, traffic accident rate, psychiatric incidence rate, occupational disease incidence rate, tumor incidence rate, and cardiovascular disease incidence rate, which are shown by data availability. Here, the income of psychiatric medical institutions, psychiatric medical institutions, occupational medical institutions, oncology medical institutions, and the number of cardiovascular patients are taken as the proxy variables of the incidence of psychiatric diseases, occupational diseases, tumors, and cardiovascular diseases, respectively. Macroeconomic information collection mainly includes: output categories, such as raw coal, coke, power generation, natural gas production; Investment category: investment completion of fixed assets in different industries, investment completion of real estate development; price category, consumer price index, retail price index, wholesale price index of industrial products; consumption category, total retail sales volume of social consumer goods, consumer confidence index, etc.; Currency category: Renminbi is the same as US dollar, Euro, Yen, etc. National exchange rate, deposit or loan interest rate of different maturities, interbank lending rate, deposit reserve, M_0_, M_1_, and M_2_, ecological environment, wastewater, waste, and solid waste discharge, etc., total 74-dimensional variables. The original data are from the website of the National Bureau of Statistics, the website of the People's Bank of China, the Wine database and the China Health Statistics Yearbook.

The data frequency of this paper is quarterly, and the sample interval is selected as 2005 Q1-2017 Q2. This period basically covers the period of steady economic growth, financial crisis and recovery, sustained economic downturn and the new normal stage of the economy. Such a setting can effectively reflect the non-linear impact of China's economic growth on the health of residents under different macroeconomic backgrounds. The basic data are processed in the following order: (1) since we can only get the annual data of the health indicators, we use the quadratic spline method to convert the annual data of the health indicators into quarterly data. (2) Logarithmic processing of absolute data; (3) Seasonal processing with Census-X12; (4) Stationary test of standardized sequence with ADF rule without time trend term and intercept term, and first-order difference processing for non-stationary sequence; (5) Data processing above is a standard time series with mean value of 0 and standard deviation of 1. Table 1 shows descriptive statistics of key variables in the model.

**Table 1:** Descriptive statistics of key variables.

**Variable name**	**Variable meaning**	**Mean value**	**Median value**	**Standard deviation**	**Maximum value**	**Minimum value**
GDP	China's economic growth	9.309091	8.098701	2.482420	14.54182	6.467350
M	Mortality rate	7.032308	7.098281	0.184227	7.167813	6.358125
INM	Infectious disease mortality rate	1.108354	1.143359	0.186766	1.465625	0.751063
ME	Medical expenditure of residents	6.409124	6.389991	0.343598	7.018347	5.847612
IFM	Infant mortality rate	12.03615	12.12500	3.784469	19.65313	6.467813
TRA	Traffic accident rate	2.517749	2.507427	0.021671	2.570678	2.496100
MD	Mental illness rate	14.42963	14.54800	0.690913	15.48258	13.27340
TUM	Tumor mortality rate	14.77865	14.84542	0.693899	15.73876	13.57095
OD	Occupational disease rate	10.95118	10.96842	0.578461	11.96419	9.679680
CD	Cardiovascular diseases rate	10.16806	10.27019	0.116526	10.28047	10.03805

### Analysis of Empirical Results

**Figure 2:**
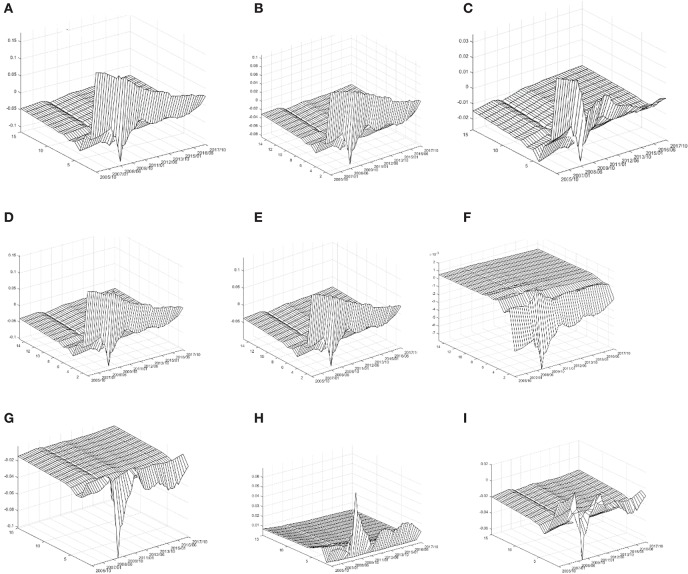
Time-varying impact of economic growth on health of residents. **(A)** Mortality rate; **(B)** infectious disease mortality rate; **(C)** medical expenditure of residents; **(D)** traffic accident rate; **(E)** infant mortality rate; **(F)** cardiovascular diseases rate; **(G)** mental illness rate; **(H)** occupational disease rate; and **(I)** tumor incidence rate.

Figure 2 series reports the impulse response on the indicators of the health of residents to economic growth. On the whole, given the positive impact of a standard unit GDP, the health indicators selected in this paper have obvious impulse response value, which indicates that China's economic growth has an impact on the health of residents. This conclusion is consistent with previous research conclusions. Secondly, at different times, the indicators of the health of residents have a pulse on economic growth. The impulse response values are obviously different, which indicates that the impact of China's economic growth on the health of residents shows obvious time-varying characteristics under different macroeconomic backgrounds. This is a new discovery of this paper, and also a good confirmation of the test results of the DCC-GARCH model. Finally, the time-varying effects of different types of the health indicators are on economic growth the difference of impulse response values implies that the impact of China's economic growth on the health of residents is heterogeneous.

Specifically, as far as the overall mortality rate is concerned, given a standard unit of positive GDP impact, M's impulse response value is basically above zero at the present time. After three periods of lag, M's impulse response value converges to zero basically, which indicates that China's economic growth has caused the simultaneous increase in population mortality rate. From the time-varying characteristics of the current period, the results show that the economic growth of China has caused a simultaneous increase in population mortality rate. During this period, the M-impulse response value showed a significant downward trend. During the period of 2013 Q3-2017 Q4, the M-impulse response value was basically below 0.05. These empirical results imply that during the sample period, the increase of population mortality caused by China's economic growth is gradually decreasing, and this decline has entered a new normal stage in China's economy. In addition, it is noteworthy that during the 2008 Q2-2009 Q3 periods, the M-impulse response value with two lags appeared to be partially negative; indicating that during the global financial crisis, after two quarters, China's economic growth reversely reduced the population mortality rate. The three-dimensional characteristics of the impulse response of INM, TRA, and IFM to GDP are similar to that of M. That is to say, China's economic growth is a standard unit. The incidence of infectious diseases rate, neonatal mortality rate, and traffic accident rate are rising at the same time, and in the sample period, this relationship shows a significant weakening trend.

As far as medical expenditure is concerned, given the positive impact of a standard unit on GDP, the impulse response value of ME in the current period is more significant. During the period of 2005 Q1-2008 Q2, the impulse response value of ME is basically around 0.03, and then it begins to decrease significantly. During the period of 2008 Q2-2009 Q3, the impulse response value of ME lagging 2 periods appears to be locally negative. During the period of 009Q3-2013 Q2, the impulse response value of ME in the current period appeared “hump” while during the period of 2013 Q3-2017 Q2, the basic value of ME in the current period was below 0.

It shows that China's economic growth reduces the incidence of cardiovascular disease rate. From the time-varying characteristics, the negative impulse response value of CD is obvious in the sample period from 2005 Q1 to 2010 Q4, especially in the period of 2008 Q2-2009 Q3, which is the most significant, while in the period of 2011 Q1-2017 Q4, the negative impulse response value of CD forms the trend of convergence to zero.

As far as the incidence of mental disease rate is concerned, given a standard unit of positive GDP impact, the impulse response value of MD is basically below 0. According to the time-varying characteristics, the impulse response value of MD is basically near 0 during the period of 2005 Q1-2008 Q1, which means that during this period, the impact of China's economic growth on the incidence of mental disease is not significant, but in the period of 2008 Q2-2009 Q3. Meanwhile, the negative impulse response value of MD was the most significant; indicating that during the financial crisis, China's economic growth significantly reduced the incidence of mental disease. During the period of 2009 Q4-2017 Q4, the negative impulse response value of MD was significantly reduced.

As far as the occupational disease morbidity rate is concerned, given a standard unit GDP positive impact, the OD impulse response value is basically above 0, which indicates that China's economic growth has led to the same upward rise in the incidence of occupational diseases rate. From the time-varying characteristics, the OD positive impulse response value is slightly >0 in the period of 2005 Q1-2008 Q1 and obvious in the period of 2008 Q2-2009 Q3. The OD impulse response value is about 0.02 during the period of 2011 Q1-2017 Q4, which indicates that the incidence of occupational disease morbidity rate increases by 0.02% for every 1% increase in GDP.

In terms of tumor incidence rate, given a standard unit GDP positive impact, the TUM impulse response values coexist in the sample period. The TUM impulse response values were significantly positive during 2005 Q1-2008 Q2 and significantly negative during 2008 Q2-2009 Q3. During 2009 Q4-2012 Q2, the TUM impulse response values turned positive and presented positively. The TUM impulse response value was around −0.02 during the period of 2012 Q3-2017 Q4.

### Research Conclusion

According to the stage characteristics of China's macroeconomic development, we divide the sample interval into four stages: stable economic growth period (2005–2008), financial crisis period (2008–2010), sustained downward economic period (2011–2013) and new normal economic situation (2013–2017). Based on the above empirical results, we draw the following conclusions: (1) During the period of steady economic growth, China's economic growth has led to a significant increase in population mortality rate, incidence of infectious diseases rate, medical expenses of residents, traffic accident rate, neonatal mortality rate, and tumor incidence rate, but has a reverse effect on cardiovascular morbidity rate, mental illness incidence rate, and occupational disease morbidity rate. In the same direction, influence is relatively weak. (2) During the financial crisis, the positive effects of economic growth on population mortality rate, incidence of infectious diseases rate, traffic accidents rate, and neonatal mortality rate were significantly reduced, while the positive effects on medical expenditure of residents, tumor incidence rate, cardiovascular incidence rate, and mental illness incidence rate were significantly inhibited, but significantly increased. The incidence of the occupational disease mortality rate was increased. (3) During the period of sustained economic downturn, the positive effects of economic growth on overall mortality rate, incidence of infectious mortality rate, traffic accident rate and neonatal mortality rate continued to decrease, while the negative effects on mental illness rate and cardiovascular morbidity rate remained. The negative effects on medical expenses of residents, occupational disease rate, and tumor morbidity rate were also observed. The positive influence of the formation degree is relatively low. (4) In the new normal stage of the economy, the positive effects of economic growth on overall mortality rate, incidence of infectious diseases morbidity rate, traffic accidents rate, and neonatal mortality rate have been further reduced, the promotion effects on occupational diseases rate and medical expenditure of residents have been significantly reduced, and the weak inhibitory effects on the formation of tumor morbidity rate have been observed, but the negative effects on neonatal mortality rate have not been observed. The inhibitory effects of cardiovascular and psychiatric morbidity were weakened. The above conclusions provide useful guidance for improving the quality of China's economic development. At the same time, using these conclusions, China's medical resources can be allocated and adjusted effectively.

### Research Limitations and Innovations

Limitations: (1) China is a super-populous country with a population of about 1.4 billion, sharing 34 provinces and autonomous regions, which are constrained by the econometric model. This paper discusses the impact of economic growth on the health of residents from the macro-overall perspective based on only technical time-series data and does not give the characteristics of regional differences of such impact. (2) It has been shown that time opportunity cost, medical payment guarantee, and ecological environment are the main transmission channels of the impact of economic development on the health of residents. The present value of data availability is partly used to complete the transmission mechanism variables (such as tobacco and alcohol consumption index, suicide rate, etc.) which are not included in the model, which is slightly regrettable.

Innovations: (1) At present, few papers are devoted to the impact of China's economic growth on the health of residents. As the world's manufacturing factory and the largest developing country in the world, this study is a supplement and enrichment to the existing relevant literature. (2) China is currently carrying out structural reform on the supply side, in which high-quality economic development is an important direction for the future of China's economy. This study has certain guiding values for further improving the evaluation system of China's high-quality economic development. (3) From the existing literature, scholars mostly use simply fixed coefficient multiple regression model to reveal the impact of economic factors on the health of residents. However, in reality, there is a lagging dynamic relationship between model variables, and with the change of macroeconomic environment, this dynamic relationship may contain non-linear characteristics. In this paper, a time-varying FAVAR model with high-dimensional variables is set up, which can effectively capture the non-linear dynamic impact of economic growth indicators on the health of residents indicators, thus reflecting the innovation of research methods.

### Future Research Directions

In future studies, in view of the differences in regional economic development, this paper will set up a non-linear panel econometric model to further empirically analyze the impact of economic growth in different provinces or autonomous regions on the health of residents.

## Data Availability Statement

The datasets generated for this study are available on request to the corresponding author.

## Author Contributions

FL advised the study and revised the draft. All authors designed and conducted the study, analyzed the data, wrote the draft, contributed to the interpretation of results, critically reviewed the draft, and approved the final manuscript.

### Conflict of Interest

The authors declare that the research was conducted in the absence of any commercial or financial relationships that could be construed as a potential conflict of interest.

## Footnotes

^1^GDP year-on-year as an agent index of economic growth, the overall population mortality rate as an agent index of health of residents, the specific data sources see below.

^2^Bernanke et al. (28) believes that the traditional VAR model can only contain 6–8 variables at most; otherwise there will be “dimension curse” problem.
